# Ibrutinib directly reduces CD8+T cell exhaustion independent of BTK

**DOI:** 10.3389/fimmu.2023.1201415

**Published:** 2023-09-12

**Authors:** Ling Li, Manzhi Zhao, Caoimhe H. Kiernan, Melisa D. Castro Eiro, Marjan van Meurs, Inge Brouwers-Haspels, Merel E. P. Wilmsen, Dwin G. B. Grashof, Harmen J. G. van de Werken, Rudi W. Hendriks, Yvonne M. Mueller, Peter D. Katsikis

**Affiliations:** ^1^ Department of Immunology, Erasmus University Medical Center, Rotterdam, Netherlands; ^2^ Cancer Computational Biology Center, Erasmus Medical Center (MC) Cancer Institute, Erasmus University Medical Center, Rotterdam, Netherlands; ^3^ Department of Pulmonary Medicine, Erasmus University Medical Center, Rotterdam, Netherlands

**Keywords:** immune checkpoint blockade, T-cell exhaustion, ibrutinib, BTK inhibitor, CD8+ T cells

## Abstract

**Introduction:**

Cytotoxic CD8+ T cell (CTL) exhaustion is a dysfunctional state of T cells triggered by persistent antigen stimulation, with the characteristics of increased inhibitory receptors, impaired cytokine production and a distinct transcriptional profile. Evidence from immune checkpoint blockade therapy supports that reversing T cell exhaustion is a promising strategy in cancer treatment. Ibrutinib, is a potent inhibitor of BTK, which has been approved for the treatment of chronic lymphocytic leukemia. Previous studies have reported improved function of T cells in ibrutinib long-term treated patients but the mechanism remains unclear. We investigated whether ibrutinib directly acts on CD8+ T cells and reinvigorates exhausted CTLs.

**Methods:**

We used an established *in vitro* CTL exhaustion system to examine whether ibrutinib can directly ameliorate T cell exhaustion. Changes in inhibitory receptors, transcription factors, cytokine production and killing capacity of ibrutinib-treated exhausted CTLs were detected by flow cytometry. RNA-seq was performed to study transcriptional changes in these cells. Btk deficient mice were used to confirm that the effect of ibrutinib was independent of BTK expression.

**Results:**

We found that ibrutinib reduced exhaustion-related features of CTLs in an *in vitro* CTL exhaustion system. These changes included decreased inhibitory receptor expression, enhanced cytokine production, and downregulation of the transcription factor TOX with upregulation of TCF1. RNA-seq further confirmed that ibrutinib directly reduced the exhaustion-related transcriptional profile of these cells. Importantly, using btk deficient mice we showed the effect of ibrutinib was independent of BTK expression, and therefore mediated by one of its other targets.

**Discussion:**

Our study demonstrates that ibrutinib directly ameliorates CTL exhaustion, and provides evidence for its synergistic use with cancer immunotherapy.

## 1 Introduction

Cytotoxic CD8+ T cells (CTLs) are the core components of adaptive immune response, which eliminate virus-infected cells and malignant cells. Following T cell receptor (TCR) engagement, CD8+ T cells become activated and exert their cytotoxic effects. Persistent antigenic stimulation induces CTL to be dysfunctional (1–3), often referred to as exhausted, and CTL immune responses are inhibited by the upregulated PD-1 and other inhibitory receptors (IRs) (4). Therefore, reducing T cell exhaustion can be a means to improve antigen-specific immunity improve chronic infection and tumor control.

Ibrutinib is an irreversible Bruton’s tyrosine kinase (BTK) inhibitor, which is approved by FDA for the treatment of B cell malignancies (5). In chronic lymphocytic leukemia (CLL), BTK is upregulated, contributing to a continuously activated BCR signaling pathway. Direct inhibition of BTK suppresses malignant B cell proliferation, therefore, impedes disease deterioration (6). Intriguingly, accumulating evidence suggest that it also affects T cells (7, 8). CD8+ T cells in CLL patients exhibit T cell exhaustion signatures, including the co-expression of inhibitory receptors and the loss of effector function (9). However, T cells in ibrutinib treated CLL patients have reduced PD-1 expression and improved cytokine production, indicating possible effects on T cell exhaustion (10). Ibrutinib treatment also reduces chronic activation markers, like CD39 and HLA-DR, on T cells in leukemia patients. Furthermore, ibrutinib improves CD19 chimeric antigen receptor T-cell (CAR T cell) viability and expansion, decreases PD-1 expression on CAR T cells, and this is accompanied by improved clinical responses (11–13). Nevertheless, the mechanism by which ibrutinib affects T cells in hematopoietic tumors remains obscure. This effect could be either direct or indirect. Since chronic antigen stimulation is a driving force of T cell exhaustion (1, 2), ibrutinib could be reducing chronic antigen stimulation of T cells by acting on malignant B cells and reducing tumor load, and thus indirectly affecting T cell exhaustion. On the other hand, ibrutinib can also indirectly affect T cell exhaustion by reducing PD-L1 expression in CLL (14) or other immune cells, such as myeloid cells that also express BTK (15, 16), and thus indirectly mitigate T cell exhaustion. The question remains, however, whether in addition to indirect effects on T cells, ibrutinib can deliver direct effects on T cells despite BTK not being expressed in T cells (13, 17).

In this study, we investigated whether ibrutinib can directly improve the characteristics of T cell exhaustion. We used our established *in vitro* CTL exhaustion induction system, in which only purified CD8+ T cells are employed, to study that ibrutinib acts directly on T cells to ameliorate exhaustion. Our data shows that ibrutinib directly reduces CD8+T cell exhaustion, while its effect is independent of BTK expression. Ibrutinib notably mitigates the transcriptional profile of exhaustion in *in vitro* induced exhausted CTLs. Our findings provide the first evidence that ibrutinib can act directly on T cells to reduce T cell exhaustion and suggests its use to support cancer immunotherapy.

## 2 Results

### 2.1 Ibrutinib reverses the exhaustion-related phenotype of *in vitro* exhausted CTLs

**Figure 1:**
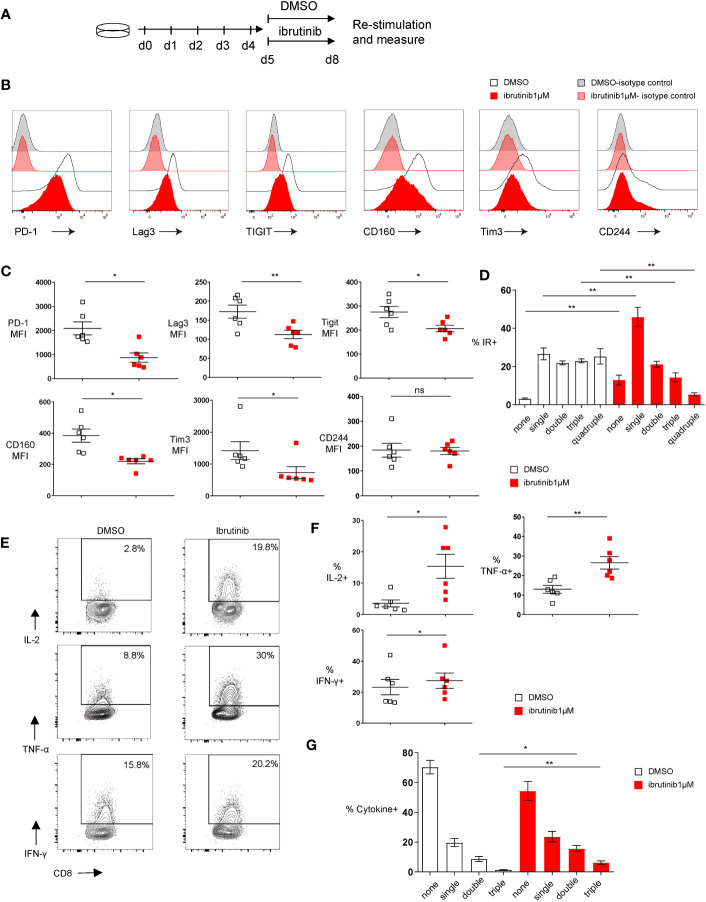
Ibrutinib rejuvenates *in vitro* exhausted CD8+ T cell functions. **(A)** Scheme of testing ibrutinib effects on *in vitro* CTL exhaustion. By repeatedly stimulating the purified OT-І T cells with OVA(257-264) peptide for 5 days, *in vitro* exhausted CTLs were induced. From day 5, the cells were treated with DMSO or 1μM ibrutinib. On day 8, function and phenotype of the cells were determined. **(B)** Representative histograms depicting the expression of inhibitory receptors and isotype controls on DMSO or ibrutinib treated exhausted cells on day 8 of the *in vitro* exhaustion culture. **(C)** Pooled data showing the median fluorescence intensity (MFI) of the inhibitory receptors expressed on DMSO or ibrutinib treated cells. **(D)** Bar chart depicting frequency of cells expressing either one, two, three or four of the inhibitory receptors (IRs) PD-1, Lag3, Tim-3 and Tigit. **(E)** Representative FACS plots illustrating percentage of cytokine-producing CD8+ T cells after OVA(257-264) peptide re-stimulation and intracellular cytokine staining. Exhausted OT-I CD8+ T cells were re-stimulated on day 8 for 6 hours with OVA(257-264) peptide and intracellular cytokines were measured by flow cytometry. **(F)** Pooled data showing the frequency of cytokine producing CD8+ T cells. **(G)** Bar graph depicting the frequency of cells producing either one, two or three of the cytokines (IL-2, TNF-α and IFN-γ) simultaneously. Each symbol represents one animal (n=6), 4 independent experiments performed. Lines depict mean ± SE. Between the groups, paired-t test was performed, except for MFI of PD-1, CD160 and Tim3, where the Wilcoxon matched-pairs signed rank test was used “ns” indicates not significant (p>0.05) *P<0.05, **P<0.01.

**Figure 2:**
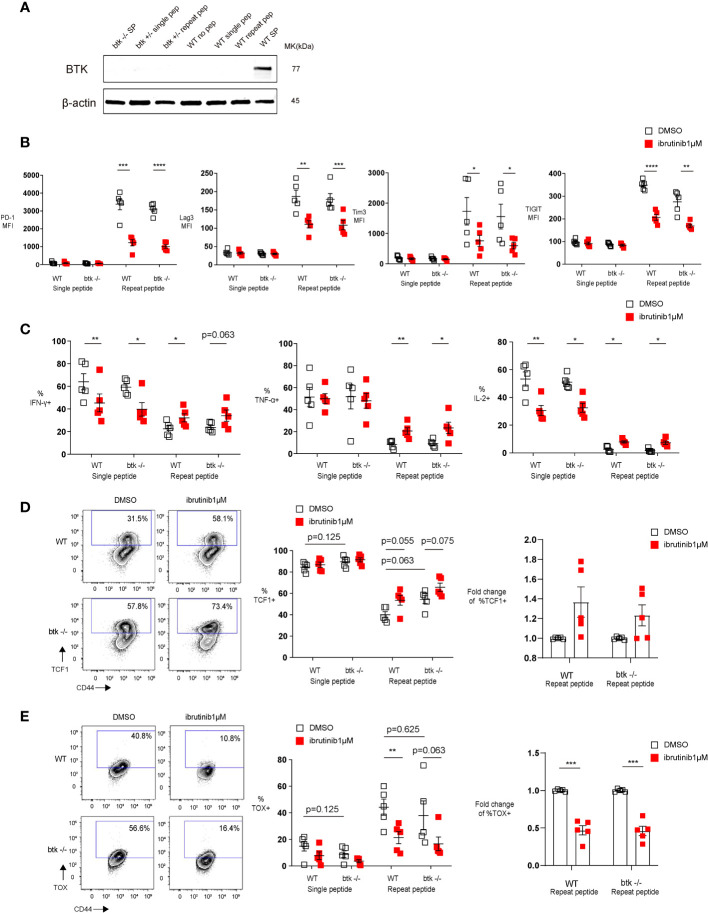
The T cell exhaustion decreasing effect of Ibrutinib is independent of Btk expression. Purified OT-І cells from wild type (WT) or btk deficient (btk-/-) mice were stimulated once with OVA (257-264) peptide or repeatedly stimulated for 5 days, and then treated with DMSO or 1 µM ibrutinib till day 8, when phenotype and function of cells was determined. **(A)** Western blot examining the expression of BTK in *in vitro* exhausted OT-I cells (day 5 of repeat peptide stimulation) or OT-I cells stimulated once with peptide. Wild type (WT) and btk+/- OT-I cells shown. WT and btk-/- splenocytes shown as positive and negative controls. **(B)** Pooled data depicting the expression of inhibitory receptors. **(C)** Pooled data showing the frequency of cytokine producing cells re-stimulated on day 8 with OVA(257-264) peptide for 6h. **(D)** Representative flow cytometry plots (left) and pooled data (middle) showing the frequency of TCF1+ CD44+ CD8+ T cells from either WT or btk-/- mice after DMSO or 1 µM ibrutinib treatment. Bar graph (right) demonstrates the fold change of %TCF1+ in exhausted T cells from either WT or btk-/- mice after treatment with DMSO or 1 µM ibrutinib. **(E)** Representative flow cytometry plots (left)and pooled data (middle) illustrating the frequency of TOX+ CD44+ CD8+ T cells from either WT or btk-/- mice after treatment with DMSO or 1 µM ibrutinib. Bar graph (right) demonstrates the fold change of %TOX+ in exhausted T cells from either WT or btk-/- mice after treatment with DMSO or 1 µM ibrutinib. Each symbol represents one animal (n=5), 5 independent experiments performed. Lines depict mean ± SE. To compare different groups, paired-t test was performed with exemption of **(B)** % of IL-2+ cells and C) % of TOX+ cells, where Wilcoxon matched-pairs signed rank test was used. *P<0.05, **P<0.01, ***P<0.001, ****P<0.0001.

To examine whether ibrutinib can directly ameliorate T cell exhaustion, we used the *in vitro* exhaustion system we established (18). In this system, purified OVA-specific OT-І transgenic CD8+ T cells that are driven to exhaustion in 5 days by repeatedly stimulating with OVA_(257-264)_ peptides (18). On day 5, ibrutinib was incubated with these exhausted T cells for an additional 3 days, and then cells were analyzed for exhaustion characteristics (**Figure 1A**). We found that 1 µM ibrutinib treatment lowered the expression of inhibitory receptors, including PD-1, Lag3, Tigit, CD160 and Tim3 (**Figures 1B, C**, and **Supplementary Figure 1**). Cells expressing multiple inhibitory receptors were also decreased with ibrutinib treatment (**Figure 1D**). Ibrutinib was able to decrease exhaustion markers in a dose-dependent manner down to 0.1 µM (**Supplementary Figure 1**). Cytokine production after peptide re-stimulation improved with ibrutinib treatment, and there were more cells producing IL-2, TNF-α and IFN-γ (**Figures 1E, F**). When the simultaneous production of these three cytokines was analyzed, the frequency of double and triple cytokine producing cells was significantly increased upon ibrutinib treatment (**Figure 1G**), which indicates that ibrutinib treatment partially rescues the polyfunctionality of exhausted T cells. Ibrutinib increased the CD107a expression and degranulation after peptide stimulation in exhausted T cells but decreased intracellular granzyme B (GzmB) (**Supplementary Figures 2A, B**). Despite increasing CD107a expression, however, ibrutinib did not improve the killing capacity of these cells (**Supplementary Figure 2C**). We next examined TCF1 and TOX, key transcription factors known to be involved in T cell exhaustion. TCF1 expression indicates progenitor exhausted T cells and serves as a predictor of ICB efficiency (19, 20). TOX increases dramatically and sustainably when T cells become terminally exhausted (21). With ibrutinib treatment, we found that the exhaustion-related transcription factor signatures of the exhausted cells improved, as it decreased TOX expression while increasing TCF1 expression in OT-І cells (**Figures 2D, E**).

To exclude the effect of ibrutinib acting by reversible binding to other kinases because of its continuous presence in our cultures, we tested drug washouts and daily ibrutinib redosing for 1 hour in our *in vitro* exhaustion system. From day 5 when cells are exhausted, cells are either treated daily (days 5, 6 and 7) with 1 µM ibrutinib or DMSO for 1 hour followed by two washing steps. However, these cells showed a partial reduction of exhaustion even in the absence of ibrutinib treatment. Ibrutinib in this scheme provided no additional phenotypic changes compared to the control (**Supplementary Figure 3**). The above indicate that the continuous TCR stimulation is required for the effect of ibrutinib in the system as this washout approach removed peptide stimulation that otherwise persists in these cultures throughout the culture period.

Overall, these results indicated that ibrutinib directly acts on T cells to ameliorate key features of CTL exhaustion by downregulating inhibitory receptors, improving cytokine production and polyfunctionality while downregulating TOX and increasing TCF1 transcription factors.

### 2.2 Reduction of CD8+T cell exhaustion by ibrutinib is not mediated by BTK inhibition

Since T cells do not express BTK (22, 23) and in order to understand how ibrutinib directly reverse exhaustion, we first performed a Western blot analysis to examine BTK expression in *in vitro* exhausted T cells. We found that BTK is not expressed in exhausted T cells (**Figure 2A**), indicating that the effect of ibrutinib on exhausted T cells is BTK-independent. To further exclude a role for BTK in ibrutinib’s effect, we submitted *btk*-/- OT-I cells to our *in vitro* exhaustion protocol and found that *btk*-/- OT-I cells, similar to wild type OT-I cells, were readily exhausted *in vitro.* Furthermore, when *btk-*/- OT-I cells were *in vitro* exhausted and treated with ibrutinib, ibrutinib retained its ability to ameliorate exhaustion-related features of these cells. Ibrutinib could decrease inhibitory receptor expression (**Figure 2B**) as well as improve cytokine production in *btk*-/- OT-I cells (**Figure 2C**). Ibrutinib also prevented the upregulation of TOX in *in vitro* exhausted *btk*-/- OT-I cells (**Figure 2E**) and a higher frequency of *in vitro* exhausted *btk*-/- T cells maintained TCF1 expression (**Figure 2D**).

Taken together, these results demonstrated that ibrutinib’s *in vitro* ability to directly reverse key features of CTL exhaustion is independent of BTK.

### 2.3 Chronic TCR stimulation in exhausted CTLs is suppressed by ibrutinib

**Figure 3:**
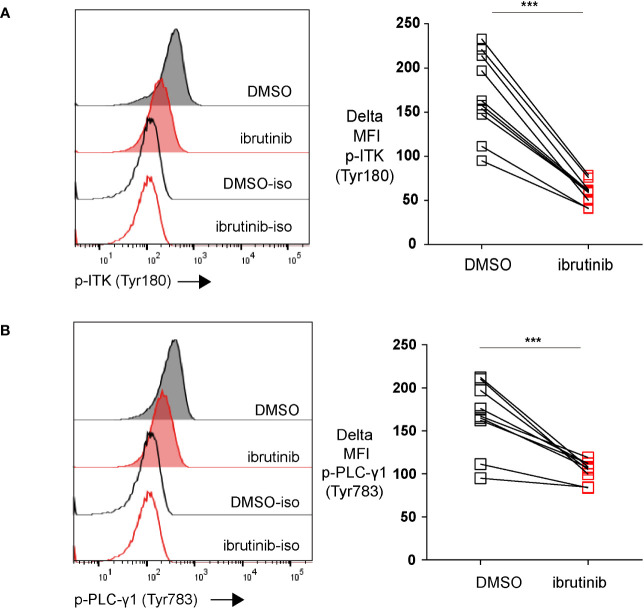
Ibrutinib downregulates the phosphorylation of both ITK (Tyr180) and PLC-γ1 (Tyr783). **(A)** Representative histograms and pooled data showing ITK (Tyr180) phosphorylation in exhausted CTLs that were treated on day 5 with ibrutinib or DMSO and detected on day 6. **(B)** Representative histograms and pooled data showing the phosphorylation of PLC-γ1 (Tyr783) in exhausted CTLs that were treated on day 5 with ibrutinib or DMSO and examined on day 6. Each symbol represents one animal (n=8), 8 independent experiments performed. Between the groups, paired-t test was performed to test for statistical significance. ***P<0.001.

CTLs become functionally exhausted in the course of chronic infections, which is induced by continuous TCR stimulation due to chronic antigen stimulation (1). In the downstream signaling of TCR, Tec family kinases are involved in the activation process. The activation of Tec kinases phosphorylates the important downstream target phospholipase C-γ1 (PLC-γ1), initiating the crucial biological cascade of TCR signaling. IL-2–inducible kinase (ITK) is the predominant Tec kinase, which is also one of the targets of ibrutinib (24). Since chronic TCR signaling stimulation drives exhaustion (1, 2), we examined whether ibrutinib affected TCR signaling in *in vitro* exhausted CTLs. Using our *in vitro* exhaustion system, we first exhausted T cells with repeat peptide stimulation. On day 5, the cells were treated with either DMSO or ibrutinib. Treating with ibrutinib inhibited the activation of ITK as the phosphorylation of ITK (Tyr180) was significantly decreased in exhausted T cells (**Figure 3A**). Furthermore, the phosphorylation of PLC-γ1 (Tyr783) was also diminished with ibrutinib treatment in these cells (**Figure 3B**). Our data indicate that the reversal of T cell exhaustion by ibrutinib, is at least in part, due to temper continuous TCR stimulation in exhausted CTLs.

### 2.4 Ibrutinib improves the transcriptional profile of *in vitro* exhausted CD8+ T cells

**Figure 4:**
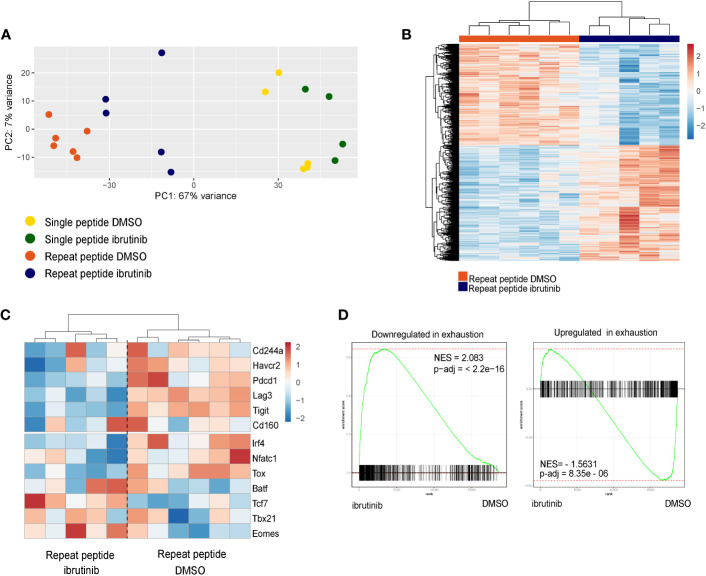
Ibrutinib reduces the exhaustion-related transcriptional signature of *in vitro* exhausted cells. RNAseq was performed with the single peptide stimulated and repeat peptide stimulated cells after treatment of cells with ibrutinib or DMSO from day 5 to day 8. **(A)** Principle Component Analysis (PCA) plot of RNAseq results. **(B)** Heatmap depicting the significantly differentially expressed genes between ibrutinib-treated cells and DMSO-treated exhausted T cells. **(C)** Heatmap showing a specific subset of exhaustion-related genes (inhibitory receptors and Transcription factors). The color key in heat maps of **(B, C)** indicates transcript-based z-score expression values. **(D)** Differentially expressed genes expression changes induced by ibrutinib in exhausted T cells were analyzed using Gene set enrichment analysis (GSEA). The gene set downregulated in exhausted T cell is enriched in genes upregulated by ibrutinib (left) while the gene set upregulated in exhausted T cell is enriched in genes downregulated by ibrutinib (right).

We next examined whether ibrutinib treatment of *in vitro* exhausted CD8+ T cells could directly change the transcriptional profile of these cells. For this we first exhausted OT-I cells for 5 days and then treated cells with ibrutinib or left them without treatment until day 8. Cells were then analyzed by RNA-seq. By visualizing the RNA-seq results with Principal Component Analysis (PCA), we found that single peptide stimulated cells and repeat peptide stimulated cells were distinctly separated (**Figure 4A**), something we have previously demonstrated (18). Ibrutinib treatment drove the repeat peptide stimulated cells to cluster more closely with single peptide stimulated cells. There were 1745 significant differentially expressed genes between the repeat peptide stimulated exhausted cells with or without ibrutinib treatment (**Figure 4B**). Specifically, ibrutinib downregulated the expression of genes encoding for the inhibitory receptors *Pdcd1* (1.8 fold), *Lag3* (1.9 fold) and *Tigit* (2.1 fold), but did not affect *Havcr2*/*Tim3*, *Cd160* and *Cd244a* significantly (**Figure 4C**). Ibrutinib also corrected some of the transcription factor changes that are induced by T cell exhaustion. *Tox* and *Irf4* were downregulated by 1.6 and 2 fold, respectively. *Tcf7* and *Eomes* were upregulated (1.8 fold and 2.2 fold, respectively) while *Tbx21*, Batf and Nfatc1 were not affected (**Figure 4C**). We next used gene set enrichment analysis (GSEA) to confirm whether ibrutinib reduced the exhausted related transcriptional profile of *in vitro* exhausted CD8+ T cells. We found that ibrutinib treated cells downregulated genes that are upregulated in T cell exhaustion while upregulating genes that are downregulated in exhaustion (**Figure 4D**), thus mitigating the gene expression changes induced by exhaustion. Performing ingenuity pathway analysis (IPA) on the differentially expressed genes between ibrutinib treated and untreated *in vitro* exhausted CD8+ T cells, revealed activation of interferon signaling and inflammation related signaling (**Supplementary Table 1A**). At the same time, the exhaustion-associated metabolic pathways, check-point blockade pathway and cell death were all inhibited after ibrutinib treatment (**Supplementary Table 1B**). Overall, these results further support that ibrutinib acts directly on exhausted CD8+ T cells and decreases their exhaustion-associated gene transcriptional signature.

## 3 Discussion

Our study demonstrates that ibrutinib directly reverses the exhaustion-related signature of *in vitro* exhausted CTLs, and this is independent of inhibiting BTK. We show that ibrutinib not only downregulated inhibitory receptors, but also improved these cells functionally shown by increased cytokine production. Ibrutinib resulted in a less exhausted transcriptional signature with changes in key exhaustion-related transcription factors.

T cells in CLL patients are dysfunctional. Improved function of T cells in B cell malignancies treated by ibrutinib have been described in recent years (10, 13, 25). However, it remains unclear if these effects are the result of reduced antigenic load as a consequence of BTK inhibition of malignant B cells or a potential indirect effect on other immune cell populations because of on-target or off-target inhibitory effects. Since chronic antigen stimulation plays a critical role in T cell exhaustion (1, 2), reducing B cell tumor load with ibrutinib could indirectly affect T cell exhaustion. Meanwhile, other cell populations can also be targeted by ibrutinib, therefore impacting T cell indirectly. Long et al. have reported that both ibrutinib and acalabrutinib, a selective BTK inhibitor, markedly reduced PD-1 and CTLA-4 expression on T cells in CLL patients (25). As this reduction was detected in patients who just completed 2 months BTK inhibitor treatment when most of patients still had lymphocytosis, these phenotypic changes on T cells were independent of tumor load. This may be due to the on-target effects of BTK inhibitor on CLL cells affecting immune suppression (25), or on other non-T cell populations (16). When CD19-targeted CAR T cell therapy was combined with ibrutinib, it was found that ibrutinib enhanced the function and engraftment of CAR T cells (11). Although these effects on CAR-T cells could also be caused by the reduced B cell tumor burden, reduced cytokine release by ibrutinib concurrently with CAR T-cell therapy was also observed in patients who had failed ibrutinib treatment alone, suggesting a tumor-burden independent effect on bystander immune and non-immune cells (26). Additionally, the off-target effect of ibrutinib, known as BTK-independent, may also contribute to the effects on T cells. This is indicated by other studies showing that the highly specific BTK inhibitors, zanubrutinib or acalabrutinib, could not induce the same changes on T cells as ibrutinib does (25, 27, 28). Our findings support that notion that ibrutinib directly acts on T cells to reinvigorate T cell exhaustion, but *in vivo* ibrutinib treatment may also affect other cells further contributing to the improvement of T cell function.

In our *in vitro* exhaustion system, 1 μM ibrutinib was used for testing the functional and transcriptional changes on exhausted T cells. Studies have shown that 1 μM ibrutinib reduces exhaustion marker expression on CLL patient T cells *ex vivo* and protects T cells against activation induced cell death (25, 27). This 1 μM ibrutinib concentration does not completely inhibit T cells as its TCR inhibitory effects can be rescued when by a strong co-stimulatory signal (29). We report here that ibrutinib worked in a dose-dependent manner and that concentrations even down to 0.1 μM, decrease exhaustion-related phenotypes on exhausted T cells. This in agreement with previous reports that show a dose-dependent inhibition of ITK by ibrutinib (17, 28). This suggests that lower concentrations of ibrutinib such as those achieved in patients and acting for longer periods may still have direct effects on T cell exhaustion *in vivo*.

Using our T cell exhaustion system, we have shown that ibrutinib affected multiple exhaustion features directly on T cells. Ibrutinib partially restored cytokine production. However, it did not improve cytotoxicity even though it increased CD107a expression and degranulation after stimulation. This may be due to the decreased GzmB expression that we found with ibrutinib treatment or to additional defects in the killing machinery of these cells. Since T cells hierarchically progress through stages of dysfunction during exhaustion and the loss of ability to lyse target cells occurs at an early stage (30, 31), impaired cytotoxic function may follow different kinetics compared to other exhaustion features such as inhibitory receptors. Therefore, it may be that the restoration of killing capacity requires more or longer inhibition of TCR signaling compared to inhibitory receptor downregulation.

Our studies show that ibrutinib directly ameliorates CTL exhaustion in a BTK-independent manner. Ibrutinib covalently binds to the active site of BTK, but also other kinases besides BTK have the corresponding cysteine residue where ibrutinib binding occurs (32). These kinases include other kinase members of Tec family (ITK, TEC, BMX and RLK), EGFR family kinases (EGFR, HER2 and HER4), BLK and JAK3 (32). Ibrutinib has different inhibitory efficiencies on these kinases, but such binding can lead to the off-targets effects. In T cells, ITK is expressed predominantly and acts as the core mediator of TCR singling pathway. Resting lymphocyte kinase (RLK), also expressed in T cells but in relatively low levels, plays a redundant role for ITK. However, RLK fails to be blocked by ibrutinib efficiently (17, 32). Therefore, ITK inhibition by ibrutinib, which has an IC_50_ for ITK of ~4.9nM (33), may largely contribute the reversal of T cell exhaustion by ibrutinib. In support of this, we show that ITK signaling is directly inhibited by ibrutinib in exhausted CTL. Ibrutinib also binds non-covalently to kinases which do not have homology to BTK’s cysteine 481 in their binding site, such as SRC-family kinases (34). The SRC-family kinases Lck and Fyn are involve in T-cell receptor proximal signaling, and can be reversibly inhibited by ibrutinib with an IC_50_ of ~6.3 nM and 29 nM respectively (33, 35). Such non-covalent reversible inhibition can be abrogated in culture by drug washout (17, 36). To exclude such non-covalent reversible inhibition in our cultures, we performed daily ibrutinib treatments for 1 hour followed by drug washout. These studies were inconclusive as washouts removed TCR stimulation from our exhaustion system and reduced T cell exhaustion. We could not perform ibrutinib washouts combined with daily peptide restimulations in this system as daily peptide re-dosing after day 5 induces extensive activation induced cell death. Therefore, we cannot fully exclude that some ibrutinib effects that we describe may be due to reversible SRC-family kinase inhibition. Ibrutinib also inhibits MEK1/2 phosphorylation downstream of TCR signaling (37), which could also contribute to the T cell exhaustion improvement seen in our *in vitro* experiments. In line with this, an inhibitor of MEK, which suppresses MAPK-PI3K-mTOR pathways activity, has been shown to improve the anti-tumor effects of CD8+ T cells and benefit immune checkpoint blockade (ICB) therapy (38–41). This could also indicate that using ibrutinib to enhance immunotherapies may have additional benefits compared to exclusively targeting a single kinase of TCR signaling pathway.

Immunotherapeutic approaches, as ICB therapy, to revive immune response have achieved clinical benefits in several types of malignancies. However, a significant portion of patients exhibit low response rates, which beseeches the development of combination therapies that increase response rates. Ibrutinib has been reported to augment anti-PD-L1 treatment in mouse lymphoma and CLL models (29, 42). We provide evidence that this is a direct effect of ibrutinib on T cell exhaustion and, at least for a major part, through impairing chronic TCR stimulation. Nevertheless, the combination therapy of ibrutinib and anti-PD-L1 checkpoint blockade, in a phase1b/2 clinical study, showed limited antitumor activity in patients with relapsed/refractory solid tumors (43). This is possibly due to the incessant administration of ibrutinib in these patients, which may hinder efficient activation of CD8+ T cells. It also suggests that it is crucial for the success of combined therapy to find the balance between blocking the chronic antigen stimulation without impeding the activation of CTLs. This could be done with lower doses of ibrutinib or intermittent treatment.

In summary, we provide evidence that ibrutinib directly reduces CD8+T cell exhaustion. This is due to its off-target effects and is at least in part due to inhibiting chronic TCR stimulation by blocking ITK signaling. We provide a rationale for using ibrutinib in cancer immunotherapies to improve the functionality of T cells and increase the efficacy of such immunotherapies.

## 4 Methods

### 4.1 Animals

C57BL6/J Tg (TcraTcrb)1100Mjb/J (OT-I) CD45.1+ mice were In-house-bred by backcrossing C57BL/6 OT-I with B6.SJL-Ptprca Pepcb/BoyJ (CD45.1+) mice (both from Charles River France). Btk deficient (*btk*-/-) OT-I mice were generated by cross breeding OT-I CD45.1+ mice with *btk* deficient C57BL/6J (44). Mouse genotyping was performed by PCR and flow cytometry analysis. All experimental mice were kept in a certified barrier facility at Erasmus Medical Center animal facility (Erasmus Dierenexperimenteel Center, EDC). All experiments were carried out in accordance with the guidelines of the Instantie voor Dierenwelzijn (IvD). The Project Proposal (AVD101002015179) of this study was approved by CCD (Centrale Commissie Dierproeven).

### 4.2 *In vitro* CTL exhaustion induction and ibrutinib treatment

*In vitro* exhausted murine CD8+ T cells were generated as previously described (18). In brief, CD8+ T cells were isolated from single-cell suspensions of OT-I mouse splenocytes by negative selection (EasySep, Stemcell Technologies). Exhausted CTLs were induced by repeat OVA peptide _(257-264)_ (Anaspec) stimulation for five days. Cells were split on day 4. Single peptide stimulated cells were stimulated with OVA_(257-264)_ peptide once in the first 2 days and were rested for additional 3 days. On day 5, cells were analyzed by flow cytometry to evaluate their exhaustion phenotypes. From day 5, both single peptide and repeat peptide stimulated cells were treated with ibrutinib (MedChemExpress, No. HY10997) or DMSO. Repeat peptide stimulated cells were maintained with OVA peptide present from day 5 to day 8 in culture medium when treated with ibrutinib. On day 8, cells were collected for flow cytometry staining. The concentration of ibrutinib tested was between 0.1 to 1 μM, which is within range of the peak plasma concentrations (0.175 to 0.45 μM) achieved in ibrutinib treated patients during clinical trials (45). The 1 μM ibrutinib concentration is consistent with the concentration used in previous studies (25, 27) and showed no cytotoxicity in our *in vitro* cultured primary T cells.

For ibrutinib treatment with washouts and redosing, cells were treated as above from day 0 to day 4. On day 5, cells are either treated daily (days 5, 6 and 7) with 1 µM ibrutinib or DMSO for 1 hour followed by two washing steps. Repeat peptide stimulated cells were added with OVA peptide together with ibrutinib treatment on day 5 before washing steps and was hence removed from culture with washes. On day 8, cells were collected for flow cytometry staining.

### 4.3 Flow cytometry

For surface staining, cells were first washed with FACS wash (HBSS containing 3% FBS and 0.02% sodium azide) and then incubated at 4°C in the dark for 20 minutes with appropriate concentrations of fluorochrome-conjugated monoclonal antibodies and appropriate isotype controls: anti-CD8a-eFluor 450 (clone 53-6.7, eBioscience), anti-CD160-PE-CF594 (clone CNX46-3, BD Biosciences), anti-Lag3-APC (clone C9B7W, BD Biosciences), anti-CD244-PE (clone 2B4, BD Biosciences; eBio244F4, eBioscience), anti-PD-1-APC-Cy7 (clone 19F.1A12, Biolegend), anti-Tim3-PE-Cy7 (clone RMT3-23, Invitrogen), anti-TIGIT-FITC (clone GIGD7, eBioscience); anti-CD44-BV786 (clone IM7, BD Biosciences). Cells were also stained with PerCP/Cyanine5.5 labeled Annexin V to exclude apoptotic and dead cells. For Annexin V staining, all solutions and buffers included 2.5 mM calcium chloride (CaCl_2_). After incubating, cells were washed one time with FACS wash and fixed with 1% Paraformaldehyde solution (PFA).

For the intranuclear staining of transcription factors, cells were first surface stained as described above. After washing with FACS wash, cells were fixed with FoxP3 Fixation Buffer (catalogue # 005523, eBioscience) for 1 hour in the dark at 4°C. After fixation, cells were washed with Perm/Wash buffer (catalogue # 008333, eBioscience) and stained for 45 minutes at 4°C in the dark with antibodies against transcription factors: anti-Tox-PE (clone TXRX10, eBioscience), anti-TCF1-A647 (clone C63D9, Cell Signaling). Then, cells were washed twice with Perm/Wash buffer and fixed with 1% PFA. Corresponding isotype controls were included with this staining protocol.

For the intracellular cytokine staining, cells were first re-stimulated with 10µg/ml OVA_(257-264)_ SIINFEKL peptide for 6 hours at 37°C and 5% CO2 in the presence of GolgiPlug(BD Biosciences) and anti-CD107a -APC-Cy7 antibodies (clone ID4B, Biolegend). Cells were then stained with surface antigen antibodies as described above. After washing with FACS wash, cells were fixed with IC Fixation Buffer (catalogue # 88-8824, eBioscience) at 4°C overnight. The next day, cells were washed with Perm/Wash buffer and stained for intracellular cytokines for 45 minutes in the dark at 4°C. Anti-IFN-γ-APC (clone XMG1.2, eBioscience), anti-TNF-α-AF488 (clone MP6-XT22, eBioscience), anti-IL-2-PE (clone JES6-5H4, eBioscience), anti-Granzyme B-PE-Cy7 (clone NGZB, eBioscience) and appropriate isotype controls (all from eBioscience) were used for cytokine detection. After staining, cells were washed twice with Perm/Wash buffer and fixed with 1% PFA.

For the intracellular phospho-protein staining, cells were harvested and fixed immediately with pre-warmed fixation buffer (catalogue # 420801, Biolegend) for 20 minutes at 37°C. After washing with Cell Staining buffer (420201, Biolegend), cells were permeabilized with pre-chilled True-Phos™ Perm Buffer (425401, Biolegend) overnight at -20°C. The next day, cells were stained with phospho-specific antibodies with for 40 minutes at room temperature in the dark after washing. Anti-BTK Phospho (Tyr223)/ITK Phospho (Tyr180) antibody (clone A16128C, Biolegend), anti-PLCγ1 Phospho (Tyr783) antibody (clone A17025A, Biolegend) or isotype control (clone MOPC-21, Biolegend) were used for phosphoflow staining. Lastly, cells were washed twice with cell staining buffer and fixed with 1% PFA.

Samples were measured on a LSRFortessa (BD Biosciences) and analyzed with FlowJo software (Version 9.9.4, Treestar, Ashland, OR, USA).

### 4.4 Western blot assay

Btk+/- and wild type OT-I cells were harvested from *in vitro* exhaustion inducing cultures on day 5. Btk-/- and WT splenocytes were used as control. Western blot was performed as previously described (46). In brief, cell pellets were washed twice with PBS buffer and then lysed in RIPA buffer (150 mM sodium chloride, 1.0% NP-40, 0.5% sodium deoxycholate, 0.1% sodium dodecyl sulfate, 50 mM Tris, pH 8.0). The total protein concentration was determined by using the Pierce BCA Protein Assay Kit (Thermo Scientific, 23227). The same amount of proteins were loaded into a 4–15% Mini-PROTEAN TGX Gel (Bio-Rad, 456-1084) and transferred to PVDF membrane (Merck Millipore, IPVH00010). Membranes were blocked with 5% BSA in Tris-buffered saline with tween (TBST), primary antibodies were incubated overnight at 4°C in TBST as follows: Rabbit anti-Mouse BTK (Cell Signaling, clone D3H5), mouse anti-Mouse β-Actin (Cell signaling, clone 8H10D10). Membranes were washed and incubated with secondary antibodies as follows: IRDye^®^ 800CW conjugated Goat anti-Mouse IgG (LI-COR Biosciences, 926-32210) and IRDye^®^ 680RD conjugated Goat anti-Rabbit IgG (LI-COR Biosciences, 926-68071) in 3% milk/TBST for 1 hour at room temperature, and imaged using an Odyssey imaging system.

### 4.5 T cell mediated killing assay

Murine T cells were induced to be exhausted as described above. Exhausted T cells were either treated with 1 μM ibrutinib or DMSO from day 5 to day 8 for a total of 3 days. On day 8, cells were washed thoroughly and rested for 24 hours before being seeded together with AE-17 tumor cells. For the co-culturing, AE-17 cells were resuspended with cell culture medium (RPMI 1640 with 10% FBS) at 10^6^ cell/ml, then pulsed with 1μg/ml OVA_(257-264)_ peptide (Anaspec). After being incubated for 1 hour at 37˚C, cells were labelled with CellTraceTM Far Red fluorescent dye (Invitrogen™, C34564) as specific target cells. Equal numbers of un-pulsed cells were labelled with CellTrace™ CFSE fluorescent dye (Invitrogen™, C34554) as non-specific control cells. For killing assay, 10^5^ peptide-pulsed and un-pulsed AE-17 cells were seeded in 12-well plate and co-cultured with different ratios of T cells (Effector: Target ratio: 0:1, 0.5:1, 1:1, 3:1) for 18 hours. The next day, cells were harvested and stained for flow cytometry. The calculation of specific killing used the formula: % specific lysis = 100 –[Target/Non-target (in the presence of effector cells)]/[Mean of Target/Non-target (in the absence of effector cells)] x 100%.

### 4.6 RNA sequencing

Using the *in vitro* exhaustion model, single peptide stimulated and repeat peptide stimulated cells were treated from day 5 to day 8, with DMSO, Ibrutinib as described above. On day 8, 0.5-1*10^6^ live CD8+ T cells were washed with PBS and immediately lysed with TRIzol LS reagent (Life Technologies) and stored at -80°C. RNA was extracted according to manufacturer’s instructions and a bioanalyzer (Agilent) was used to determine the quality and quantity of the extracted RNA. Barcoded sequencing libraries were generated using the NEBNext Ultra II Directional RNA Library Prep Kit for Illumina (NEB #E7760S/L). mRNA was isolated from total RNA using the oligo-dT magnetic beads. After fragmentation of the mRNA, cDNA synthesis was performed. This was used for ligation with the sequencing adapters and PCR amplification of the resulting product. The quality and yield after sample preparation was measured with the Fragment Analyzer. The size of the resulting products was consistent with the expected size distribution (a broad peak between 300-500 bp). Paired-end sequencing was performed on a Hiseq2500 machine (Illumina) for 150 cycles.

Paired-end raw FASTQ files were analyzed with the nf-core/RNA-seq pipeline (v3.0) using Nextflow (v20.11.0-edge) and its default settings (47, 48). Quality of the sequencing was reported with FastQC (v11.9). Subsequently, bases with low Phred scores (<= 30) were trimmed and the reads with a low mean Phred score were removed using Trim Galore! (v6.6). Trimmed FASTQ reads were mapped to the mouse reference genome version GRCm38 and Ensembl GRCm38.81.gtf gene annotation file using RSEM (v1.3.1), which umbrellas STAR (v2.7.6a) as read aligner. Next, SAMtools (v 1.10) processed the alignment files and extracted mapping statistics for post-alignment stats (49–51). Quality of each sample alignment was visually inspected using reports derived from RSeQC (v3.0.1), Qualimap (v2.2.2-dev) and Preseq (v2.0.3), including read inner distance plots, splice junction annotations, the genomic origin of the mapped reads, and the estimated complexity of the sequencing library (52–54). RSEM estimated transcript counts were imported into R (v4.0.3), transformed to gene counts using tximport (v1.18) and analyzed with DESeq2 (v1.30.0) (55, 56). Only “protein_coding” and “lncRNA” genes were kept using biomaRt (v2.46.0) annotation (57). Gene counts were transformed using the “rlog” function of DESeq2 for visual inspection and for Principal Component Analysis (PCA) utilizing its “plotPCA” function.

### 4.7 Differential expression analysis

Differentially expressed genes were calculated using DESeq2. p-values were calculated using Wald statistical tests, and either the single peptide experiments or DMSO were defined as the reference. Fold Changes (FC) were shrunk with the DESeq2 function “lfcshrink” using method “apeglm” (58).

Genes were indicated as differentially expressed with an adjusted p-value < 0.05, calculated with the Benjamini-Hochberg multiple hypothesis testing method, a BaseMean ≥ 50 counts, and an absolute log2FC > 0.2. Heatmaps were made using the R package pheatmap (v1.0.12), Z-scores were calculated per gene on the rlog counts. Hierarchical clustering was performed using complete clustering and Euclidean distances for both the samples and genes in heatmaps with all statistical significant differentially expressed genes. In the heatmap with a subset of exhaustion-related genes the samples were clustered using the Ward algorithm with a Pearson’s correlation coefficient matrix.

Gene Set Enrichment Analysis (GSEA) was performed with the complete gene-set after shrinkage of the fold changes (59). Genes were ranked on the FC and compared with CD8+ T cell specific exhaustion gene-sets. The CD8+ T cell exhaustion gene-sets were based on the publication of Bengsch B, et al. and downloaded from PubMed Central (60). Enrichment plots were made using R package fgsea (v1.16.0) with 100.000 permutations. The GSEA was calculated separately for the upregulated and downregulated genes. Ingenuity Pathway Analysis (IPA, Qiagen) was performed on all significant differentially expressed genes using IPAs default parameters.

### 4.8 Statistics

Statistical analysis was performed by Prism software (GraphPad Prism 9, Version 9.0.0). Shapiro-Wilk normality test was used to assess the normality of the population distribution. Two-tailed, paired-t test were used to test significant differences between normally distributed populations and Wilcoxon matched-pairs signed rank test was assessed for non-normally distributed data. P values lower than 0.05 were considered statistically significant, with the numbers of asterisks in the figures indicating the p value: * P<0.05, ** P<0.01 and *** P<0.001 and **** P<0.0001.

## Data availability statement

The RNA-seq data is accessible at Gene Expression Omnibus (GEO) platform (https://www.ncbi.nlm.nih.gov/geo/), with accession number GSE220433. The RNA-seq pipeline parameters, R code, and external data is available on our departmental bitbucket: https://bitbucket.org/immunology-emc/btk_ctl_publication/.

## Ethics statement

The animal study was approved by Instantie voor Dierenwelzijn (IvD) and CCD (Centrale Commissie Dierproeven). The study was conducted in accordance with the local legislation and institutional requirements.

## Author contributions

LL, MZ, CK, MC, MW contributed to the *in vitro* experiments. MZ, LL, YM and PK contributed to data analysis. DG, HW performed the Bioinformatics analysis. Study was designed by MZ, LL, RH, YM and PK. Study was conceived by MZ, YM and PK. LL and MZ contributed equally to experimental design, execution and manuscript writing. Manuscript was written by MZ, LL, YM, DG, HW and PK. All authors contributed to the article and approved the submitted version.

## Supplementary material

Supplementary Table 1 IPA analysis depicting the canonical pathways activated **(A)** or inhibited **(B)** by treating with ibrutinib *in vitro* exhausted CTL. The percentage of molecules regulated in the pathway, the z-score and the p-value are shown.

Supplementary Figure 1 Ibrutinib reduce inhibitory receptors expression in *in vitro* exhausted CD8+ T cells in a dose-dependent manner. Pooled data showing the MFI of the inhibitory receptors including PD-1, Tigit, Tim3, Lag3 and CD160 expressed on exhausted T cells treated with different concentrations of ibrutinib. Each symbol represents one animal (n=8), 4 independent experiments performed. Between the groups, Wilcoxon matched-pairs test was performed to test for statistical significance. *P<0.05, **P<0.01

Supplementary Figure 2 Ibrutinib did not improve cytotoxic function of exhausted T cells despite improving degranulation. Followed *in vitro* 5 day exhaustion assay protocol, the cells were treated with DMSO or 1 μM ibrutinib from day 5 to day 8. On day 8, cells were stained for GzmB or stimulated with peptide and CD107a expression was determined. Killing assay was performed on day 9, with all the cells being washed twice on day 8. **(A)** Pooled data showing MFI of GzmB on DMSO or ibrutinib treated cells. **(B)** MFI of CD107a expression on DMSO or ibrutinib treated cells with or without OVA peptide re-stimulation depicted. Each symbol represents one animal (n=9), 5 independent experiments performed. Between the groups, Wilcoxon matched-pairs test was performed to test for statistical significance. *P<0.05, **P<0.01 **(C)** Single peptide stimulated cells or repeat peptide stimulated cells treated with ibrutinib or DMSO from day 5 to day 8 were co-cultured with target cells at different ratios on day 9. Percentage of specific lysis is shown. Line depicts mean ± SE. Four independent experiments performed, each symbol represents 5-7 animals.

Supplementary Figure 3 Inhibitory receptors expression in *in vitro* ibrutinib-treated exhausted CD8+ T cells with or without washout and redosing steps. Pooled data showing the MFI of the inhibitory receptors including PD-1, Tigit, Tim3, Lag3 and CD160 expressed on DMSO or ibrutinib treated cells in the with or without washout and redosing. Each symbol represents one animal (n=9), 5 independent experiments performed. Between the groups, Wilcoxon matched-pairs test was performed to test for statistical significance. *P<0.05, **P<0.01.
