# Supplementary material for: Ibrutinib directly reduces CD8+T cell exhaustion independent of BTK
Source: Front Immunol. 2023 Sep 12;14:1201415. doi: 10.3389/fimmu.2023.1201415 (PMC10523025; doi:10.3389/fimmu.2023.1201415)
Supplement: Supplementary Table 1 — IPA analysis depicting the canonical pathways activated (A) or inhibited (B) by treating with ibrutinib in vitro exhausted CTL. The percentage of molecules regulated in the pathway, the z-score and the p-value are shown. [file DataSheet_1.docx]

**Tables with titles and legends**

Supplementary Table 1

| Predicted activated pathways in ibrutinib treated *in vitro* exhausted CTLs | | | | |
| --- | --- | --- | --- | --- |
| Rank | Pathways | % molecules regulated | z-score | p-value |
| 1 | Role of JAK1, JAK2 and TYK2 in Interferon Signaling | 38.5 | 2.449 | 6.17E-06 |
| 2 | iNOS Signaling | 27.7 | 2.530 | 1.70E-05 |
| 3 | Interferon Signaling | 25.0 | 1.667 | 7.59E-04 |
| 4 | Cell Cycle: G2/M DNA Damage Checkpoint Regulation | 22.0 | 1.508 | 6.61E-04 |
| 5 | TNFR2 Signaling | 21.9 | 0.816 | 6.46E-03 |
| 6 | STAT3 Pathway | 21.5 | 0.688 | 7.08E-08 |
| 7 | TNFR1 Signaling | 21.2 | 0.632 | 9.33E-04 |
| 8 | 4-1BB Signaling in T Lymphocytes | 20.6 | 1.342 | 9.12E-03 |
| 9 | Inflammasome pathway | 20.0 | 2.000 | 0.05 |
| 10 | Th1 Pathway | 18.9 | 1.091 | 1.58E-05 |

B

| Predicted inhibited pathways in ibrutinib treated *in vitro* exhausted CTLs | | | | |
| --- | --- | --- | --- | --- |
| Rank | Pathways | % molecules regulated | z-score | p-value |
| 1 | Glycolysis I | 38.5 | -3.162 | 6.17E-06 |
| 2 | Colanic Acid Building Blocks Biosynthesis | 35.7 | -1.342 | 2.14E-03 |
| 3 | Superpathway of Geranylgeranyldiphosphate Biosynthesis I (via Mevalonate) | 27.8 | -2.236 | 7.24E-03 |
| 4 | PD-1, PD-L1 cancer immunotherapy pathway | 27.4 | -1.000 | 1.82E-10 |
| 5 | Myc Mediated Apoptosis Signaling | 26.0 | -0.832 | 3.47E-05 |
| 6 | Estrogen-mediated S-phase Entry | 23.1 | -0.816 | 8.71E-03 |
| 7 | Gluconeogenesis I | 23.1 | -2.449 | 8.71E-03 |
| 8 | IL-23 Signaling Pathway | 21.7 | -1.000 | 1.26E-03 |
| 9 | p53 Signaling | 19.4 | -0.535 | 5.62E-05 |
| 10 | Death Receptor Signaling | 18.8 | -1.213 | 1.38E-04 |

**Supplementary Table 1:** IPA analysis depicting the canonical pathways activated **(A)** or inhibited **(B)** by treating with ibrutinib *in vitro* exhausted CTL. The percentage of molecules regulated in the pathway, the z-score and the p-value are shown.
