# Supplementary figures and images for: Ibrutinib directly reduces CD8+T cell exhaustion independent of BTK
Source: Front Immunol. 2023 Sep 12;14:1201415. doi: 10.3389/fimmu.2023.1201415 (PMC10523025; doi:10.3389/fimmu.2023.1201415)

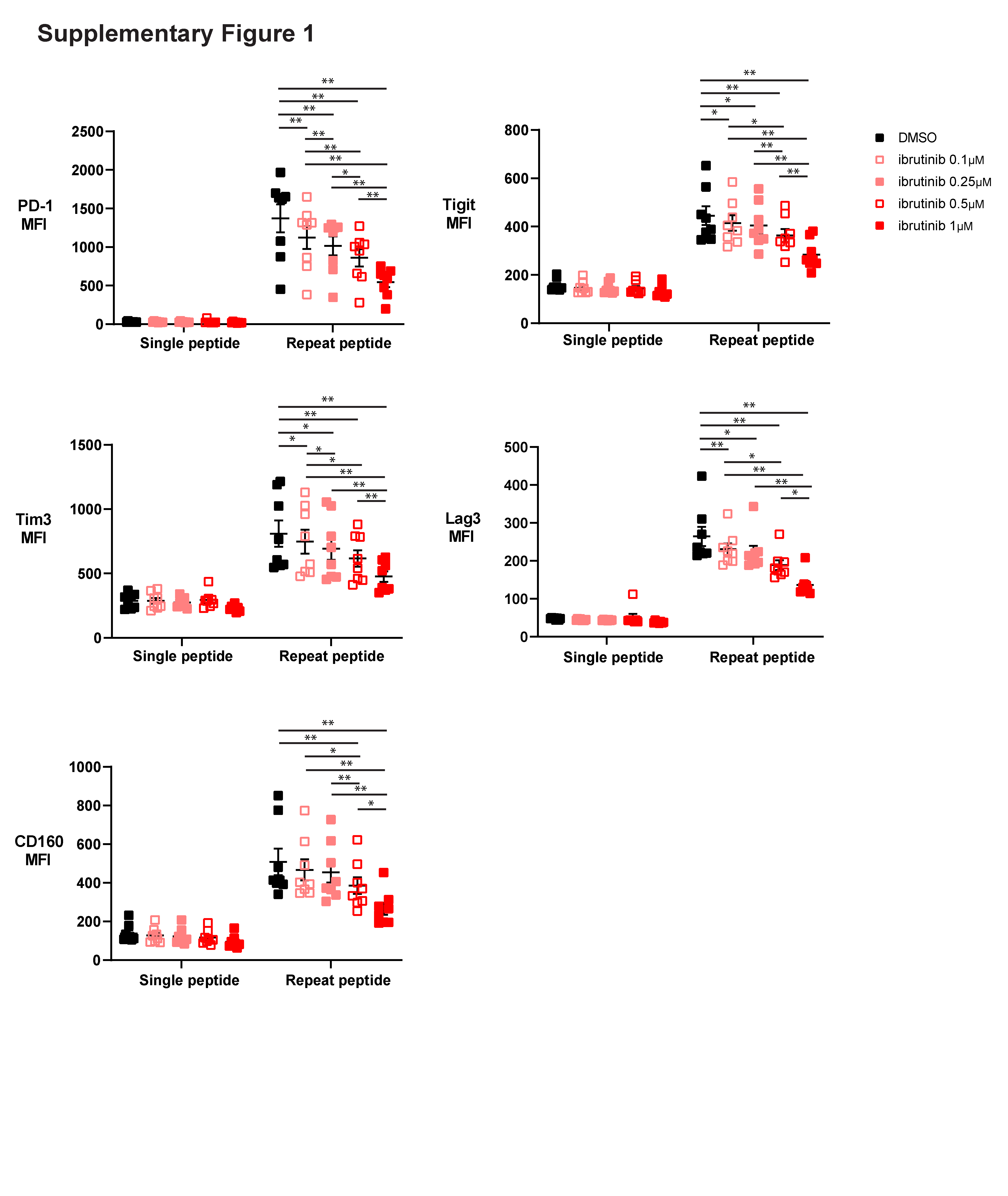

Supplement: Supplementary Figure 1 — Ibrutinib reduce inhibitory receptors expression in in vitro exhausted CD8+ T cells in a dose-dependent manner. Pooled data showing the MFI of the inhibitory receptors including PD-1, Tigit, Tim3, Lag3 and CD160 expressed on exhausted T cells treated with different concentrations of ibrutinib. Each symbol represents one animal (n=8), 4 independent experiments performed. Between the groups, Wilcoxon matched-pairs test was performed to test for statistical significance. *P<0.05, **P<0.01 [file Image_1.tif]

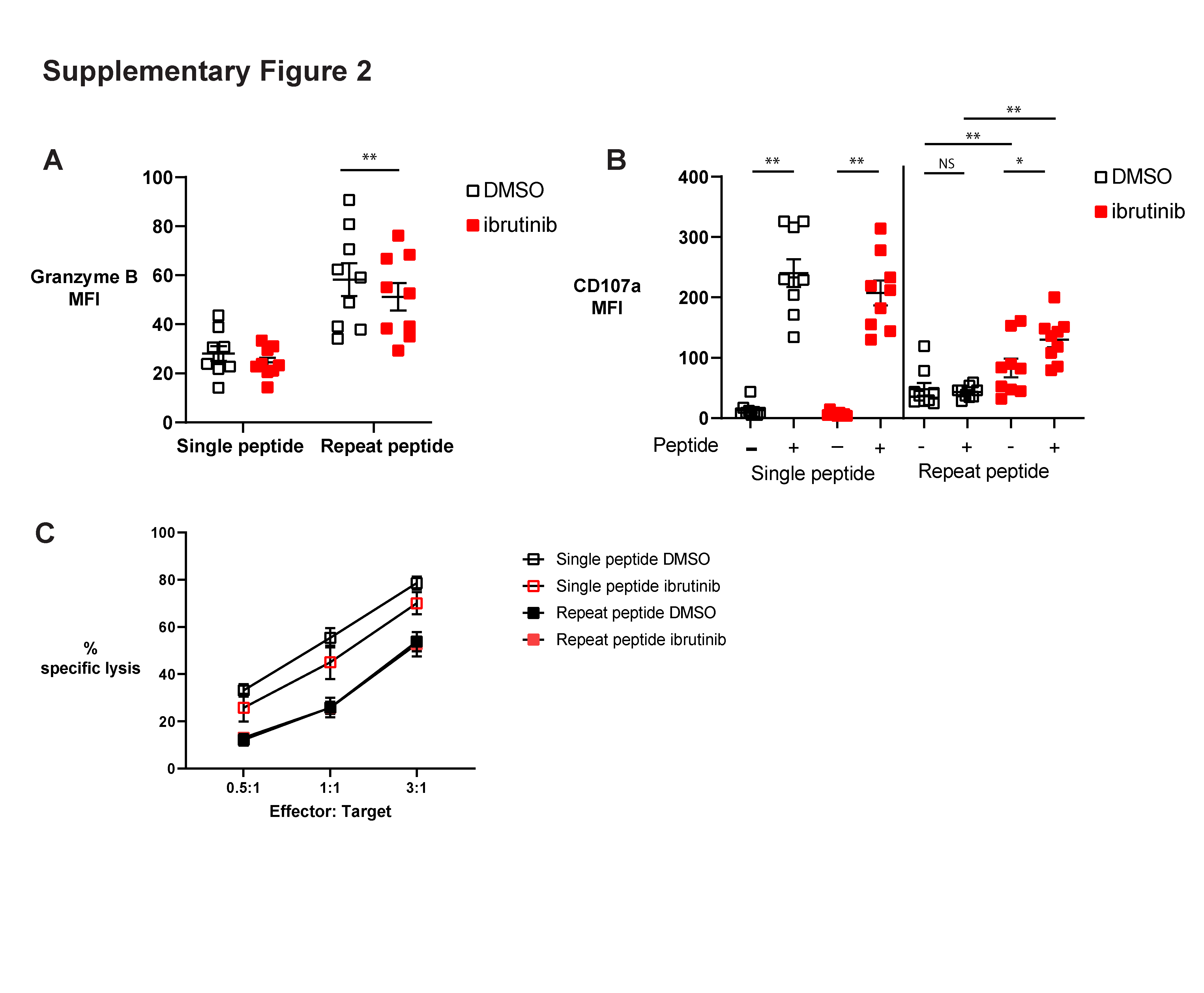

Supplement: Supplementary Figure 2 — Ibrutinib did not improve cytotoxic function of exhausted T cells despite improving degranulation. Followed in vitro 5 day exhaustion assay protocol, the cells were treated with DMSO or 1 μM ibrutinib from day 5 to day 8. On day 8, cells were stained for GzmB or stimulated with peptide and CD107a expression was determined. Killing assay was performed on day 9, with all the cells being washed twice on day 8. (A) Pooled data showing MFI of GzmB on DMSO or ibrutinib treated cells. (B) MFI of CD107a expression on DMSO or ibrutinib treated cells with or without OVA peptide re-stimulation depicted. Each symbol represents one animal (n=9), 5 independent experiments performed. Between the groups, Wilcoxon matched-pairs test was performed to test for statistical significance. *P<0.05, **P<0.01 (C) Single peptide stimulated cells or repeat peptide stimulated cells treated with ibrutinib or DMSO from day 5 to day 8 were co-cultured with target cells at different ratios on day 9. Percentage of specific lysis is shown. Line depicts mean ± SE. Four independent experiments performed, each symbol represents 5-7 animals. [file Image_2.tif]

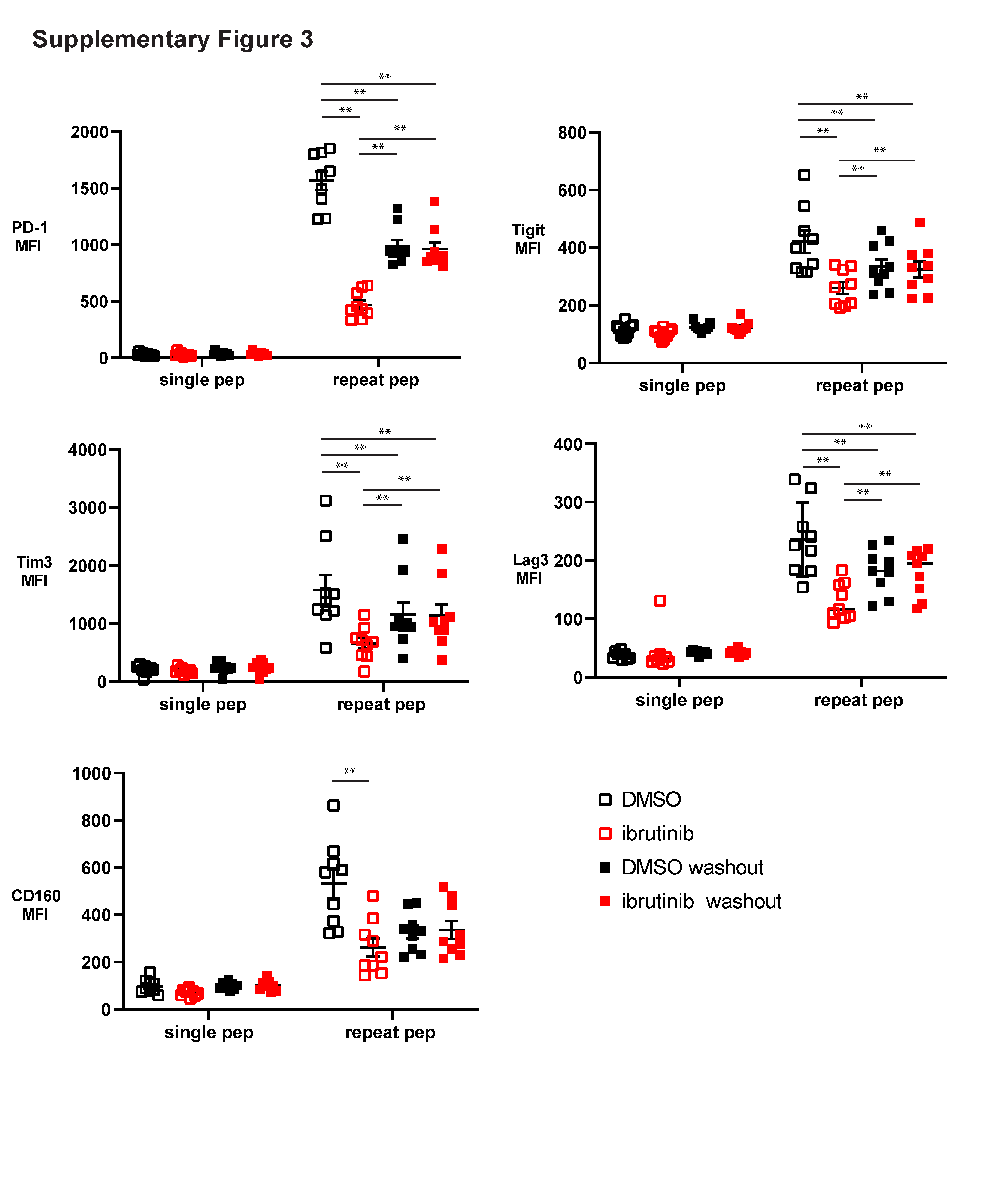

Supplement: Supplementary Figure 3 — Inhibitory receptors expression in in vitro ibrutinib-treated exhausted CD8+ T cells with or without washout and redosing steps. Pooled data showing the MFI of the inhibitory receptors including PD-1, Tigit, Tim3, Lag3 and CD160 expressed on DMSO or ibrutinib treated cells in the with or without washout and redosing. Each symbol represents one animal (n=9), 5 independent experiments performed. Between the groups, Wilcoxon matched-pairs test was performed to test for statistical significance. *P<0.05, **P<0.01. [file Image_3.tif]
